# Comparative analysis of acute and chronic corticosteroid pharmacogenomic effects in rat liver: Transcriptional dynamics and regulatory structures

**DOI:** 10.1186/1471-2105-11-515

**Published:** 2010-10-14

**Authors:** Tung T Nguyen, Richard R Almon, Debra C DuBois, William J Jusko, Ioannis P Androulakis

**Affiliations:** 1 BioMaPS Institute for Quantitative Biology, Rutgers University, Piscataway, New Jersey, USA; 2 Biomedical Engineering Department, Rutgers University, Piscataway, New Jersey, USA; 3 Chemical & Biochemical Engineering Department, Rutgers University, Piscataway, New Jersey, USA; 4 Department of Pharmaceutical Sciences, State University of New York at Buffalo, Buffalo, New York, USA; 5 Department of Biological Sciences, State University of New York at Buffalo, Buffalo, New York, USA; 6 New York State Center of Excellence in Bioinformatics and Life Sciences, Buffalo, New York, USA

## Abstract

**Background:**

Comprehensively understanding corticosteroid pharmacogenomic effects is an essential step towards an insight into the underlying molecular mechanisms for both beneficial and detrimental clinical effects. Nevertheless, even in a single tissue different methods of corticosteroid administration can induce different patterns of expression and regulatory control structures. Therefore, rich *in vivo *datasets of pharmacological time-series with two dosing regimens sampled from rat liver are examined for temporal patterns of changes in gene expression and their regulatory commonalities.

**Results:**

The study addresses two issues, including (1) identifying significant transcriptional modules coupled with dynamic expression patterns and (2) predicting relevant common transcriptional controls to better understand the underlying mechanisms of corticosteroid adverse effects. Following the orientation of meta-analysis, an extended computational approach that explores the concept of agreement matrix from consensus clustering has been proposed with the aims of identifying gene clusters that share common expression patterns across multiple dosing regimens as well as handling challenges in the analysis of microarray data from heterogeneous sources, e.g. different platforms and time-grids in this study. Six significant transcriptional modules coupled with typical patterns of expression have been identified. Functional analysis reveals that virtually all enriched functions (gene ontologies, pathways) in these modules are shown to be related to metabolic processes, implying the importance of these modules in adverse effects under the administration of corticosteroids. Relevant putative transcriptional regulators (e.g. RXRF, FKHD, SP1F) are also predicted to provide another source of information towards better understanding the complexities of expression patterns and the underlying regulatory mechanisms of those modules.

**Conclusions:**

We have proposed a framework to identify significant coexpressed clusters of genes across multiple conditions experimented from different microarray platforms, time-grids, and also tissues if applicable. Analysis on rich *in vivo *datasets of corticosteroid time-series yielded significant insights into the pharmacogenomic effects of corticosteroids, especially the relevance to metabolic side-effects. This has been illustrated through enriched metabolic functions in those transcriptional modules and the presence of GRE binding motifs in those enriched pathways, providing significant modules for further analysis on pharmacogenomic corticosteroid effects.

## Background

Glucocorticoids (GC) are a class of steroid hormones present in almost every animal cell, playing a central role in a wide range of physiological responses [1]. Because of their potent anti-inflammatory and immunosuppressive effects, synthetic glucocorticoids referred as corticosteroids (CS) (e.g. methylprednisolone - MPL) have been used widely in pharmacology as a therapeutic option for a wide range of autoimmune and inflammatory diseases [2,3]. However, beneficial effects are derived from magnifying the physiological actions of endogenous glucocorticoids, causing a variety of side effects following long-term treatment with this class of drugs e.g. hyperglycemia, dyslipidemia, arteriosclerosis, muscle wasting, and osteoporosis [4-7]. The physiological and pharmacological effects of corticosteroids are complex and manifest themselves with expression changes of many genes across multiple tissues [8-10]. It has been observed that even in a single tissue different dosing regimens of CS administration can induce different patterns of expression [11-13]. As such genes with similar expression profiles under acute CS administration may not exhibit similar expression patterns during continuous infusion, pointing to the possibility of alternative regulatory mechanisms. Therefore, a better understanding of corticosteroid pharmacogenomic effects from multiple dosing regimens are very valuable not only to reveal the transcriptional dynamics under different patterns of input perturbations but also to provide an insight into the underlying molecular mechanisms of action, for both beneficial and detrimental effects, and thus for the optimization of clinical therapies.

It has been noted that genes affected by CS include both immunosuppressive genes, mostly associated with therapeutic effects, and metabolic genes often associated with adverse effects whose regulation is mainly controlled by glucocorticoid receptor gene mediated pathways [6]. Unbound CS binds with cytosolic free glucocorticoid receptors (GR) releasing it from the heat shock complex allowing dimerization and translocation into the nucleus where it binds to glucocorticoid response element (GRE) of the target genes, leading to enhancement or inhibition of the target gene expression. As a result, long-term treatment with corticosteroids results in sustained up- or down-regulation of numerous genes, leading to a new steady state which might be the basis for occurrence of adverse effects. However, it has also been noted that chronic infusion of CS causes a sustained down-regulation of the receptor (mRNA and thus protein) [14,15]. While several alternative mechanisms have been proposed [16-18] it is still not understood why drug effects remain strong although GR mRNA is down-regulated to the point of almost being eliminated. A plausible explanation is that besides direct regulation through GRE binding sites in the 5' regulatory regions of genes, there are changes in expression that are also the indirect results of effects due to changes in expression of transcription factors (TFs) that act as secondary biosignals directly or indirectly modulating the transcription of genes [15,19,20]. Thus, along with identification of expression patterns, predicted regulatory control structures are also an essential source of information towards understanding corticosteroid effects.

In this study we address the question as to whether (1) significant transcriptional modules coupled with complex patterns of mRNA changes across multiple dosing regimens of corticosteroids and (2) their common regulatory controls can be computationally identified. Hypothetically, transcriptional modules that are significantly coexpressed under different dosing regimens will be important gene clusters for further analysis towards better understanding of both beneficial and adverse effects of corticosteroids, especially the metabolic side-effects since these patterns are survived under a long-term treatment of corticosteroids. The hypothesis explored here is that if two or more genes have the same temporal expression profiles in response to different dosing regimens, they are more likely to share some common regulatory mechanisms. The liver was selected because of its major role in both the physiological efficacious and adverse effects of corticosteroids e.g. altering the expression of serum proteins that regulate immune/inflammatory responses [21], enhancing the expression of liver enzymes involved in metabolic effects (gluconeogenesis and lipid metabolism) [22].

However, rich *in vivo *datasets of pharmacological time-series across multiple dosing regimens are often obtained from different microarray platforms and time-sets [11,23], leading to a problematical issue for computational analysis [24-26]. As an example, in a study comparing normal and chronic lymphocytic leukemia B-cells, Wang et al. [27] identified only 9 differentially expressed genes across all three studies, when combining results from three different platforms, while there are at least 1,172 differentially expressed genes in each individual platform. In general, there are two important issues relevant to the analysis of data derived from different platforms: (i) genes may be present in one platform but not in the other, and (ii) genes present on both platforms may not be represented by the same probes. Since different microarray platforms do not contain the same probesets, and even do not have a similar hardware design and sample processing protocols, standard analyses may not yield comparable expression level quantifications across platforms, leading to many challenges for computational models aiming at the analysis of microarray data from heterogeneous sources [25,28,29].

A number of approaches have been proposed and are generally classified into two main categories: (1) integrate raw expression profiles from different studies into one dataset so that available computational models can be directly applied, and (2) develop and/or utilize a unitless statistic as a primary analysis and then combine the result through a meta-level analysis. The former category can be further divided into two sub-classes, namely combining raw data through a normalization and/or transformation procedure [30-33] and pooling raw information from common probes that can be mapped to the same Unigene clusters or full-length mRNA transcripts [34-37]. However, these approaches are not general enough to make data from different platforms fully compatible [25,38]. Since combining data across different platforms remains a serious challenge, meta-analysis - the second category - has been identified as a more popular technique in order to combine results, and thus data, from a number of independent studies [39,40]. The assumption here is that while the raw expression levels from different platforms may not be comparable, the results of the primary analysis should be. However, almost all prior studies has focused on the discovery of genes that are differentially expressed in conjunction with standard models such as effect size models [41-43], Bayesian models [44,45].

Consequently, in order to identify significant clusters of genes that share common expression patterns across multiple dosing regimens, we extend our prior study [46] in the aspect of (i) producing an agreement matrix (AM) that describes the agreement levels of co-expression of genes across multiple conditions and (ii) successively searching clusterable subsets to infer all such gene clusters. The approach follows the concept of meta-analysis to avoid the limitation of incompatible data across multiple datasets from different platforms (also different tissues, time-grids, as well as lab-protocols when applicable). The unitless statistic, expressing the confidence level of co-expression is the agreement level of cluster assignments drawn from multiple clustering runs. There remain a number of open critical issues associated with a single clustering run (e.g. the input number of clusters [47,48], the biases and assumptions of distance metrics and/or clustering methods [49], cluster significance [50]), and thus consensus clustering coupled with the examination of AM distribution has been designed with the aims of reducing aforementioned limitations [46,51]. Once the AM is obtained for each condition independently (e.g. each dosing regimen in this case), an average agreement matrix is calculated to estimate the confidence levels of coexpression between genes across multiple conditions, thus combining data from different datasets into a single input for the next analysis. For the analysis at the meta-level, we extend the selection and clustering processes (also proposed in [46]) to identify all possible clusters of genes that are highly coexpressed with the average AM above as the input. As such these clusters of genes will share common patterns of expression across multiple dosing regimens. Additionally, due to the selection of all possible patterns of expression several clusters may have similar expression patterns and thus we also provide a heuristic as an optional procedure to merge such similar clusters based on a criterion of maximizing the total homogeneity and separation of selected clusters. Subsequently, we analyze promoter regions of genes in every cluster in order to predict putative transcriptional regulators, aiming at providing another source of information towards better understanding those complex patterns of expression and the underlying regulatory mechanisms of corticosteroid effects.

Our results demonstrate that the proposed computational approach is highly effective on both synthetic and real data. When applying the approach to real time-series datasets (acute/chronic corticosteroid administration [11,23]), selected patterns of transcriptional responses are enriched in a biological sense with relevant putative-regulatory controls and significant metabolic pathways in each pattern. Computational results are further validated predicated upon literature evidence.

## Methods

### 1. Datasets

#### Synthetic data

A number of synthetic datasets from the open literature are utilized to assess our approach for finding common sets of genes that are highly coexpressed across multiple conditions. Specifically, we used a series of four high-noise 20-timepoint sine-format synthetic datasets with different number of replicates at each time-point (1, 3, 4, and 20 respectively) from [52,53]. Each dataset contain five separate sets with 400 genes allocated equally in 6 classes, each of which contains the same list of genes but has different patterns across five conditions. For each set, in the first step the data are generated according to an artificial pattern Φ(i, t, l) which shows the values for gene i at time-point t in cluster l; four of six clusters follow the sine function i.e. Φ (i, t, l) = sin(2πt/10 - w_l_) (w_l _is some random phase shift between 0 and 2π), and the other two follow the non-periodic linear function (Φ (i, t, 5) = t/20 and Φ(i, t, 6) = -t/20), i = 1,...,400, t = 1,...,20, l = 1,...,4. In the second step, let x(i, t, r) be the error-added value for gene i, time-point t and repeat; x(i, t, r) is randomly drawn from a normal distribution N(μ, σ) where μ is the value of the synthetic pattern Φ(i, t, l) and σ is equal to λσit (σit is randomly extracted from measurement errors observed in the yeast galactose data [54] and λ is the multiplicative factor that controls the noise level). High-noise synthetic data are generated with λ = 6 [53]. Datasets are downloaded from the links provided in synthetic data in Additional File 1.

#### Acute corticosteroid administration

Forty-seven male ADX Wistar rats weighting from 225 to 250 g underwent right jugular vein cannulation under light ether anesthesia 1 day before the study [23]. Forty-three rats were injected with a single intravenous bolus dose of methylprednisolone (MPL) of 50 mg/kg. Animals were sacrificed by exsanguinations under anesthesia and liver samples were harvested at 0.25, 0.5, 0.75, 1, 2, 4, 5, 5.5, 6, 7, 8, 12, 18, 30, 48, and 72 after dosing. The sampling time points were selected based on preliminary studies describing GR dynamics and enzyme induction in liver. Four untreated rats were sacrificed at random times and nominally considered as 0 h controls. The gene expression was obtained via the Affymetrix RG-U34A array which consists of 8,799 probesets. The data are publicly available through the GEO Omnibus Database under the accession number GDS253.

#### Chronic corticosteroid administration

In a similar experiment model, forty rats were given 0.3 mg/kg/hr infusions of MPL over 168 h via an Azlet pump [11]. The pump drug solutions were prepared for each rat based on its predose body weight. Animals were sacrificed at various times up to 7 days; specifically the time-points included are 6, 10, 13, 18, 24, 36, 48, 72, 96, and 168 h. A control group of four animals was implanted with a saline-filled pump and killed at various times throughout the 7-day study period. Unlike the previous experiment, the microarray platform for this dataset is the RAE230A which consists of 15,923 probesets. The data are publicly available through the GEO Omnibus Database under the accession number GDS972.

All protocols followed the Principles of Laboratory Animal Care (Institute of Laboratory Animal Resources, 1996) and were approved by the University at Buffalo Institutional Animal Care and Use Committee.

### 2. Identifying critical transcriptional modules

**Figure 1:**
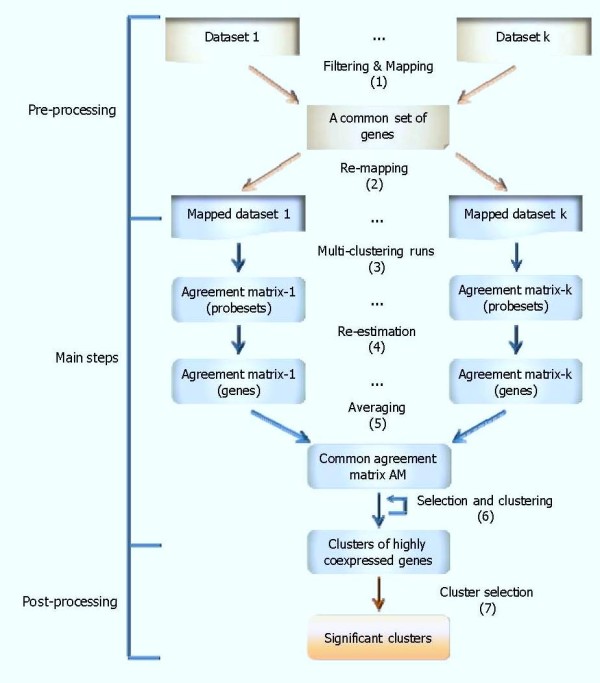
**The flowchart of the approach**. The pre-processing section refers to filtering for differentially expressed probesets in each dataset, mapping to gene symbols to extract a set of common genes that are present across all datasets, and then re-mapping to corresponding probesets in each particular dataset. The main steps include establishing the AM to characterize how much confidence two probesets (and two genes) are co-expressed in each condition (and then across all conditions) and searching for all possible clusters of co-expressed genes based on the common AM. The post-processing step will select those clusters that are significant and optionally merge those with similar expression patterns if indicated.

The general computational problem can be briefly defined as follows. We are given a set of N genes $G={\left\{g_{i}\right\}}_{i=1}^{N}$ and K conditions. For each condition k, every gene is characterized by one or more time-series expression profiles with R_ki _corresponding probesets over T_k _time-points $G_{k}={\left\{g_{ki}\right\}}_{i=1}^{N},g_{ki}=\left\{g_{ki}^{r},r\in R_{ki}\right\},g_{ki}^{r}={\left\{g_{kit}^{r}\right\}}_{t=1}^{T_{k}},k=1,...,K$. The question then becomes to search for clusters of genes that are highly coexpressed across all K conditions with a confidence level δ. The term 'highly coexpressed' is used in the sense that $\forall g_{i},g_{j}\in C,\frac{1}{K}\sum_{k=1}^{K}P_{k}\left(g_{i}\wedge g_{j}\right)\geq \delta$ where C denotes a, yet to be determined, cluster and *P_k_*(*g_i _*∧ *g_j_*) is the confidence level that two gene profiles i and j are clustered together in condition k; a gene profile includes sets of corresponding probesets R_ki _of gene i in condition k, *k *= 1,...,*K*. The subscripts {i, j}, t, k, r indicate the {gene id}, time, condition, and probesets respectively. It should be also noted that in this work, we used three different terms to refer to the same object (e.g. a set of genes that are coexpressed across multiple conditions): 'cluster' when designing the algorithm, 'pattern' when exhibiting the expression changes, and 'module' when charactering the biological function. The framework contains several step displayed in Figure 1.

#### The pre-processing step

Each dataset is pre-filtered to identify differentially expressed probesets. Since we would like to identify gene clusters with common expression patterns across multiple conditions, input datasets must contain the same set of genes. Thus using the respective platform information, probesets in each dataset are mapped to a list of genes and then the intersection across those gene lists is evaluated to extract a common set of genes which are differentially expressed across multiple conditions (i.e. datasets). However, genes are sometimes characterized by multiple probesets whose expression profiles may be similar or sometimes different, but not identical. These probesets can be considered as replicates of expression profiles for a gene and thus taking an average expression profiles across all these probesets to characterize for the expression profile of the gene may lose useful potential information. Therefore, from the common set of genes we re-map genes to corresponding probesets in each dataset with the respective platform before starting the analysis.

#### Construction of the agreement matrix

**Figure 2:**
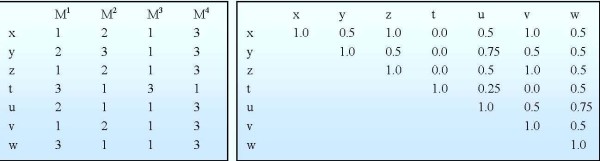
**An example of the primary clustering results (left) and the agreement matrix**. The left is the summary results of running m clustering times (m = 4 in this example, represented by M^1^...M^4^) with nc* = 3 initial clusters on N objects (N = 7, represented by x, y, z...). The right shows the corresponding agreement matrix whose entry M_xy _is the probability that two corresponding objects are clustered together by M^1^,...,M^4^.

The *agreement matrix *(AM) quantifies the likelihood that two objects (x, y) are assigned to the same cluster (Figure 2). If m clustering runs are performed on the data, each entry (termed 'agreement level') will show the frequency with which two objects are assigned to the same cluster over 'm' clustering runs. The AM entries are defined as follows:

(1) $$\begin{array}{l} M_{xy}=\frac{1}{m}\sum_{h=1}^{m}M^{\left(h\right)}(x,y)\\ where\;M^{\left(h\right)}(x,y)=\left\{ \begin{array}{l} 1\;if\;x\;and\;y\;are\;clustered\;together\;when\;running\;method\;M^{\left(h\right)}\\ 0\;otherwise \end{array}\right. \end{array}$$

In addition to the various clustering methods that were utilized, different distance metrics (Euclidean, Pearson correlation, and Manhattan [55]) are also explored in order to attenuate the biases associated with individual distance metrics. In our implementation, we are using hierarchical clustering (hclust), divisive analysis clustering (diana), fuzzy analysis clustering (fanny), partitioning around medoid (pam) with Pearson correlation and Manhattan metric, k-means (kmeans), fuzzy c-means (cmeans), self-organizing map (som), and model-based clustering (mclust) with Euclidean metric as the core clustering methods (supported by R packages) [56-61]. Since clustering results are highly dependent on the input number of clusters (nc), the sensitivity of the AM as a function of nc was examined to find a 'suggestive' number of clusters (nc*) for each particular dataset. After identifying nc* based on the procedure in our prior work [46], all clustering runs are repeated with nc* to produce the final AM for further analysis (see more details in [46]).

If two probesets (x, y) are clustered together, it is implied that their expression profiles are similar under a specific condition k. Therefore, the fraction of times (M_xy_) they are clustered together over multiple clustering runs can be considered as the confidence level that they are coexpressed since M_xy_, by construction, aims at eliminating method-specific biases and assumptions. Subsequently, we calculate the average agreement levels between sets of corresponding probesets of any two genes to estimate the confidence level that those two genes are coexpressed in a specific condition. The AM entries in condition k is re-estimated as follows

(2) $$AM_{ij}^{\left(k\right)}=\frac{1}{\left|R_{ki}\right|\left|R_{kj}\right|}\sum_{x\in R_{ki}}\sum_{y\in R_{kj}}M_{xy},i,j=1,...,N$$

With the assumption that the unitless statistics, i.e. the confidence level of co-expression, is comparable across multiple conditions and different platforms [37], we estimate the confidence level of co-expression between two genes across multiple conditions by taking the average. While combining raw data remain challenges, the estimation of a unitless statistics provides a simple but efficient combination of heterogeneous data for further analyses.

(3) $$AM_{ij}=\frac{1}{K}\sum_{k=1}^{K}AM_{ij}^{\left(k\right)},i,j=1,...,N$$

As a result, we obtain an agreement matrix whose entries exhibit a quantity that shows how confident genes are coexpressed. This will be the input for the selection and clustering process.

#### Selection and clustering

With the hypothesis that the more clusterable the data is the more biologically relevant it is, we applied our previously proposed procedure to select a more 'hypothetically clusterable' subset from the entire set of genes [46]. The main hypothesis underlying the selection is that AM entries associated with genes at the 'hypothetical' core of an expression pattern (or a cluster) will be consistently grouped together over multiple clustering runs. This should be manifested by high corresponding values in the AM, whereas genes belonging to the 'hypothetical' core of two clearly distinct clusters are associated with consistently low AM entries. On the contrary, cluster assignments associated with genes at cluster boundaries or between clusters would be very sensitive to the method used and thus they would have relatively moderate agreement levels with other genes. As a result, with a user-defined confidence level δ genes associated with moderate AM entries ($1-\delta <AM_{ij}^{\left(k\right)}<\delta$) are eliminated to produce a more 'clusterable' subset of genes (δ = 70% in this study). The process starts removing genes associated with the highest number of moderate AM entries and then updates the AM for the next loop until no moderate AM entry exists. The corresponding subset of genes is now considered as a 'hypothetically clusterable' subset since any two genes are highly coexpressed or non-coexpressed with the confidence level at least δ. Subsequently, we used the concept of consensus clustering [51,62,63] to divide the subset of genes into a number of clusters by applying the hierarchical clustering with the selected AM as input data. The algorithm starts with every gene filling a cluster and then grouping two clusters into a new one for each loop so that any pair of genes belonging to a new cluster always has an agreement level greater than or equal to δ. The iteration is stopped when no more new cluster is formed (see more details in [46]).

**Figure 3:**
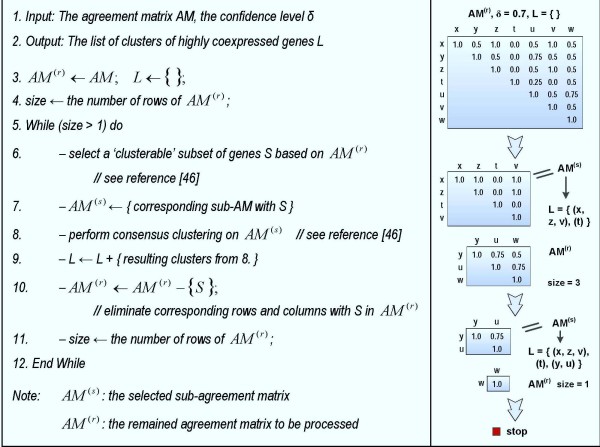
**The integrating clustering and selection procedure**. The left panel depicts a pseudo-code description of the algorithmic procedure. The right panel illustrates an example of the process. At iteration 1, the algorithm selects a 'clusterable' subset of genes including (x, z, t, v) that results in two clusters (x, z, v) and (t). The remained AM consists of corresponding rows and columns of genes (y, u, w) from the original AM. At iteration 2, the procedure selects (y, u) and the remained AM now contains only one gene (w); at that point, the process terminates.

However, since there should be existed clusters of genes located closely to other clusters in the data and the input number of clusters for the core analysis is only a suggestive one, those clusters may not be completely separated. As a result, although genes that belong to those clusters are identified as highly coexpressed, their relationship to genes in other clusters cannot be uniquely determined. Therefore, some significant clusters may be not included in the selected subset due to the constraint of 'clusterable' selection. Since we would like to obtain all significant patterns of expression, the procedure of selection and clustering is repeated on the removed domain. The removed domain consists of a set of unselected genes whose coexpression levels are still high as quantified agreement levels in the original AM. After extracting the sub-agreement matrix corresponding to the set of unselected genes, the entire process of selection and clustering is applied again with the same confidence level δ as before. The procedure is reiterated until no more clusters of genes are recognized. Figure 3 presents the pseudo-code of the procedure and an example to illustrate the process.

**Figure 4:**
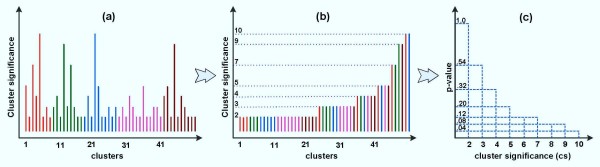
**Estimating the cluster significance threshold given a user-defined p-value**. An illustrating example is shown in which n_r _= 5 random data are generated, the data are subsequently clustered according the proposed clustering/selection procedure and cluster significance distribution are depicted in (a) and (b) following sorting. The corresponding p-value for each cluster significance cs is estimated and depicted in (c). Thus, given a p-value, we can infer the corresponding cluster significance threshold. For example, for a p-value = 0.05, all clusters with cluster significance ≥ 10 are selected and if p-value = 0.1, all clusters with cluster significance ≥ 8 are considered as significant clusters.

Furthermore, due to the nature of clustering, trivial clusters may be identified in the final results. In order to exclude such trivial clusters, each cluster C is assigned with a simple hypothetical quantity called 'cluster significance' which represents how large the cluster is in this study. We then create the distribution of cluster significance on random data to estimate the cluster significance threshold corresponding to a user-defined threshold p-value for cluster selection. Without loss of generality we select K = 1 for the random data and assume that each probeset in the mapped dataset D correspond to a gene. The suggestive number of clusters nc* for D is searched with the process in [46]. Subsequently, D is randomly resampled (permutation plus convex-hull [50]) a number of times (n_r_), for each of which the entire process starting from building the AM with the same nc* to extracting clusters of highly coexpressed genes is repeated and the resulting random clusters are returned. After that, the procedure estimates the cluster significance, which is simply the cluster-size in this study, for these random clusters and constructs a distribution of cluster significance. The corresponding p-value of cluster significance cs is defined as the number of random clusters whose significance is greater than cs over the total number of clusters identified in n_r _resamples: $pvalue\;\left(cs\right)=\frac{\sum clusters\;with\;cluster\;significance\geq cs}{\sum random\;clusters}$. As a result, given a threshold p-value (p-value = 0.05 in this study), the corresponding cluster significance threshold is inferred (Figure 4) and only clusters with significance greater than this threshold are selected.

#### Merging similar patterns

Because of the nature of the approach, it is quite reasonable to expect that the clustering process can break out patterns of expression into several sub-patterns. Thus, we repeat the process on the eliminated domains to extract all possible significant clusters, resulting in that several clusters may have a similar expression pattern but are separated into two or more clusters. Because cluster homogeneity reflects the similarity of expression profiles within a given cluster and cluster separation quantifies how effectively expression profiles are discriminated, we provide an optional heuristic in order to merge similar patterns together according to the criterion of maximizing sum of homogeneity and separation of all final output clusters. Starting with all significantly selected clusters, the procedure searches for a combination of two similar patterns so that their combination will generate a maximal increase of the sum of homogeneity and separation of all current clusters after merging those two patterns. The process is repeated until no more combinations are found i.e. any new combination always reduces the sum of homogeneity and separation. Eventually, a list of significant expression patterns that characterize the underlying transcriptional responses is generated. The metric used during the evaluation of the heuristic is quantified as follows:

(4) $$\begin{array}{l} \underset{C}{\max}\left\{homogeneity+separation\right\}=\underset{C}{\max}\left\{\frac{1}{K\;n}\sum_{k}\sum_{p}H_{k}(C_{p})+\frac{2}{K\;n\left(n-1\right)}\sum_{k}\sum_{p<q}S_{k}\left(C_{p},C_{q}\right)\right\};\\ H_{k}(C_{p})=\frac{\sum_{g_{i},g_{j}\in C_{p};g_{i}\neq g_{j}}sim\left(g_{ki},g_{kj}\right)}{||C_{p}||\left(||C_{p}||-1\right)};S_{k}(C_{p},C_{q})=\frac{\sum_{g_{i}\in C_{k};g_{j}\in C_{l}}dis\left(g_{ki},g_{kj}\right)}{\left||C_{p}||\;\;||C_{q}|\right|};\\ sim(g_{ki},g_{kj})=\frac{1}{\left\|R_{ki}\right\|\left\|R_{kj}\right\|}similarity\left(g_{ki}^{r},g_{kj}^{r'}\right);dis\left(g_{ki},g_{kj}\right)=\frac{1}{\left\|R_{ki}\right\|\;\left\|R_{kj}\right\|}dissimilarity\left(g_{ki}^{r},g_{kj}^{r'}\right);\\ p=1,...,n,q=1,...,n,k=1,...,K,r\in R_{ki},r'\in R_{kj} \end{array}$$

where C is the current set of selected clusters $C={\left\{C_{p}\right\}}_{p=1}^{n}$ and n is the current number of clusters; *H_k _*(*C_p_*) is the homogeneity of cluster C_p _in condition k and *S_k _*(*C_p_*, *C_q_*) is the separation between cluster C_p _and C_q _in condition k; *sim*(*g_ki_*, *g_kj_*) and *dis*(*g_ki_*, *g_kj_*) are the average similarity and dissimilarity (or distance) respectively between all probesets of gene i and gene j in condition k. Similarity is measured by the Pearson correlation coefficient and dissimilarity is estimated by the Pearson correlation distance.

### 3. Predicting putative transcriptional regulators

#### Promoter identification

Promoters of genes are extracted from a rich database of promoter information with a default length (500 bp upstream and 100 bp downstream of the transcription start site) if there is no experimentally defined length as suggested by Genomatix [64]. In order to identify putative transcriptional regulators, we explore the basic underlying assumption of comparative genomics which states that functional regions evolve in a constrained fashion and thus at a lower rate than non-functional regions [65,66]. It implies that conserved regions in a set of orthologous sequences survive due to their special functional implications i.e. TFBSs located on these conserved regions will be more promising as functional binding sites and thus associated TF families are more relevant to our context. Therefore, each promoter is characterized by a set of promoters from orthologous genes of other vertebrate species, if available (e.g. *Homo sapiens*, *Mus musculus, Macaca mulatta, Pan troglodytes, Equus caballus, Bos Taurus, Gallus gallus, etc*.). To be consistent in the search for conserved regions on promoter sequences in order to identify putative transcription factor binding sites (TFBSs) we eliminate those that do not consist of more than two orthologous promoters.

#### Putative functional binding sites

**Figure 5:**
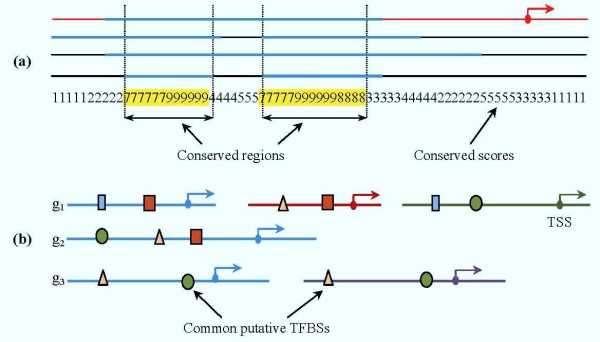
**Identification of promoter conserved regions and common physical TFBSs**. (a) Estimation of conserved regions on a single promoter (the red one) based on Dialign's alignment scores from a set of orthologous promoters. (b) Finding common physical TFBSs accounting for the case that genes may have multiple alternative promoters. TFBSs present on the conserved regions of any alternative promoter of a gene are also considered as putative TFBSs for that gene.

In order to identify conserved regions for each promoter DIALIGN [67] was used to perform multiple sequence alignments with the input sequences including each sequence as well as its orthologous promoters. DIALIGN was selected because it has many applications in comparative genomics [68]. Also, a recent benchmark study for the alignment of non-coding DNA sequences has concluded that it can produce alignments with high sensitivity as well as specificity to detect constrained sites [69]. Following the alignment among orthologous promoter sequences, we relied on the conserved scores returning from DIALIGN (with the similarity threshold of diagonals or corresponding segments involved at least 5 bases) to locate conserved regions which are defined as sub-sequences that are longer than 10 bp and continuously scored greater than the average score of all the alignment's conserved scores (Figure 5a).

We next apply MatInspector [70] to scan for all physical TFBSs and only those that overlap with the conserved regions selected above are kept for further analysis. We used a common core similarity 0.75 and utilized the optimal matrix similarity threshold for each position weight matrix (a corresponding profile of TFBSs) suggested from MatBase, Genomatix [64] which ensure that a minimum number of matches are found in non-regulatory sequences i.e. the false positive matches is minimized. However, a gene may have multiple alternative promoters [71] and virtually in all cases, it is not known which promoter of the gene is activated. Therefore, all putative TFBSs detected from all alternative promoters of a gene are considered as candidates to infer putative transcriptional regulators for the gene. Subsequently, we estimate the common level of each candidate above in each corresponding module and select those TFBSs present more than a common threshold (70% in this study) (Figure 5b). Associated TF families with those selected TFBSs are inferred and considered as transcriptional regulators for corresponding transcriptional modules.

## Results and Discussion

### Method evaluation on synthetic data

In order to evaluate the effectiveness of the proposed approach, we use synthetic data with known class structure as described earlier. The process of evaluation is repeated four times with four different datasets that are created with different number of replicates for each time-point (1, 3, 4, and 20 respectively). In each time, we use five high-noise sets as the data for five conditions (K = 5), each of which has 400 genes distributed across 6 clusters; each cluster has different patterns across five conditions but has the same set of gene ids. We set the same parameters for all evaluation in this study and also for the analysis on real time-series datasets, specifically the confidence level of coexpression δ = 70% and p-value = 0.05 for the selection of significant clusters. Furthermore, the testing process on synthetic data is done without the merging option. Without loss of generality, we assume that each gene has only one probeset in this evaluation. The performance of the approach is assessed through its ability to recover the number of cluster structures and the list of gene ids identified in each cluster. We use the adjusted Rand index [53,72] which is a statistic that measures the extent of concurrence between the clustering results and the underlying known class structure to evaluate the approach's performance in identifying gene clusters that are coexpressed across multiple conditions. The larger the Rand index is, the higher the agreement between the results and prior knowledge of cluster structure. The number of selected genes, recovered cluster structures and the accuracy on the selected domain are listed in Table 1.

**Table 1:** Effectiveness of the approach on synthetic data

Synthetic data	Number of selected genes	Number of clusters	Accuracy* (Adjusted Rand-index)
Dataset 1	174/400	4	100.0%
Dataset 2	368/400	6	100.0%
Dataset 3	395/400	6	100.0%
Dataset 4	378/400	6	100.0%

*: The accuracy is only estimated on the selected domain

Due to the fact that these datasets are high-noise synthetic data, some cluster structures may be missed when there is only one measurement at each time-point. However, when the number of replicates is increased, the number of cluster structures is recovered. As discussed in our previous study [73], this is a reasonable observation due to the effect of gene expression replicates on clustering performance. Additionally, we also examine an alternative approach which is more intuitive in identifying gene clusters that are coexpressed across multiple conditions. Instead of performing a meta-analysis to avoid the limitation of incompatible data across different platforms, we can separately identify significant clusters of genes that are coexpressed in each condition (set of data) and then obtain the intersection among these gene clusters across all conditions. In this experiment, we used pam [60], mclust [57], and consensus clustering (consclust) [46] as standard single clustering methods to identify clusters in each set of data, for which nc* = 6 is the input number of clusters. We then simply took the intersection between clusters from set to set and only keep those clusters that contain more than 5 genes as significant clusters for the final estimation of accuracy. The number of selected genes, number of clusters, and accuracy on the selected domain are listed in Table 2.

**Table 2:** Effectiveness of the approach on synthetic data

	pam			mclust			consclust		
	**# of sel. genes**	**# of clusters**^**+**^	Accuracy* (%)	**# of sel. genes**	**# of clusters**	**Accuracy** **(%)**	**# of sel. genes**	**# of clusters**	**Accuracy** **(%)**

Dataset 1	122	7|6	74.8|82.8	155	9|7	87.8|84.1	68	4|4	100|100
Dataset 2	337	6|6	100|100	343	6|6	100|100	330	6|6	100|100
Dataset 3	374	6|6	100|100	380	6|6	100|100	374	6|6	100|100
Dataset 4	376	15|7	68.8|94.6	375	13|7	80.1|94.2	302	11|7	80.9|92.4

^+^: only clusters with more than 5 genes;*: the accuracy is estimated on the selected domain; the number of clusters and the accuracy are formatted as 'before merging' | 'after merging'

In general, this approach selects a smaller number of genes with an equal or greater number of cluster structures, resulting in lower accuracy. As an example, in each set of dataset 4 there are two cluster structures that are not clearly identifiable. As a result, a single clustering methods (even consensus clustering) may fail to properly separate them in each set, leading to the situation where the intersection between clusters from set to set divides those cluster structures into many sub-clusters with a small number of genes. On the contrary, by taking the average of the co-expression levels across multiple sets, the relationship of whether two genes are coexpressed across multiple conditions can be recovered. Consequently, our proposed approach is more advantage, resulting in a final highly correct classification as illustrated in Table 1. Furthermore, since this simpler alternative approach produces many resulting clusters, we also attempted to apply the proposed merging process to reduce the number of clusters as well as improve the accuracy if applicable. However, its testing performance is still not as high as that of our proposed approach although we do not apply the merging process for the proposed approach in this test. Additionally, the alternative approach is highly sensitive with the initial number of clusters. For instance, when we constantly set nc* = 7 and test on dataset 3, without the merging option our approach still recovers the correct number of cluster structures with high accuracy: (number of selected genes, number of clusters, accuracy) = (386, 6, 100%) whereas 'pam' approach yields (366, 13, 87.8%), 'mclust' provides (360, 11, 82.3%), and 'consclust' does (351, 7, 98.3%). Since this information is not available for real datasets, the more sensitive with it the less robust the approach is. Therefore, by taking the average of the co-expression levels between two genes across multiple datasets, our proposed approach provides more robust results.

### Acute vs. Chronic CS administration

For the analysis of corticosteroid administration, the pre-processing step (Figure 1) is performed to provide corresponding mapped datasets. The datasets are first filtered for differentially expressed probesets using ANOVA technique (p-value < 0.05) implemented in R [74] and also customized by our previous work for easy uses [46]. 2,920 probesets in the acute and 4,361 probesets in the chronic are selected for further analysis. To obtain the common set of genes across two conditions, these probesets are mapped into sets of genes based on the corresponding platform information. 2,920 differentially expressed probesets in the acute are mapped into a set of 2,340 genes and 4,361 probesets in the chronic are mapped into another set of 4,076 genes. The intersection of these two gene-sets yields 967 genes in common for both dosing regimens. From this common gene set, the re-mapping process subsequently returns a corresponding set of 1,314 probesets for the acute and a set of 1,112 probesets for the chronic data. All datasets (including synthetic data) are pre-processed with the model in our previous study to estimate the 'true' expression profiles that are integrated with potential information in replicates instead of simply taking the average expression profiles [73]. The suggestive number of clusters nc* for both datasets is 7.

Subsequently, we apply the proposed approach with the merging option to the intersection set of 967 genes that are affected by corticosteroid administration across the two dosing regimens. We obtain 6 significant clusters with 315 genes in total. These clusters are hypothesized to be transcriptional modules which share common regulatory mechanisms since they consist of genes that exhibit similar expression patterns in both acute and chronic dosing regimen. Table 3 shows the distribution of these 315 genes over six modules and also briefly describes how the pattern of expression changes. Although genes may exhibit simple or complex patterns of expression during corticosteroid administration, we crudely classify those patterns into up- or down- with one or two phases of regulation.

**Table 3:** Characterization of significant transcriptional modules

Transcriptional modules	1		2			3			4		5		6		
**Number of genes**	**97**		**45**			**34**			**71**		**14**		**54**		

Expression pattern in acute*	DD	UU	U	DD	U	D	UU	D	UU	DD	DD	UU	U	DD	U

Expression pattern in chronic*	D	UU	D	U	DD	U	UUUU	U	DD	U	DDDD		UU	DD	

*: Patterns consist of one-phase regulation (up-down/down-up), two-phase regulation (up-down-up/down-up-down) or simply up- or down-regulation. Details are listed in expression patterns in Additional File 2.

**Figure 6:**
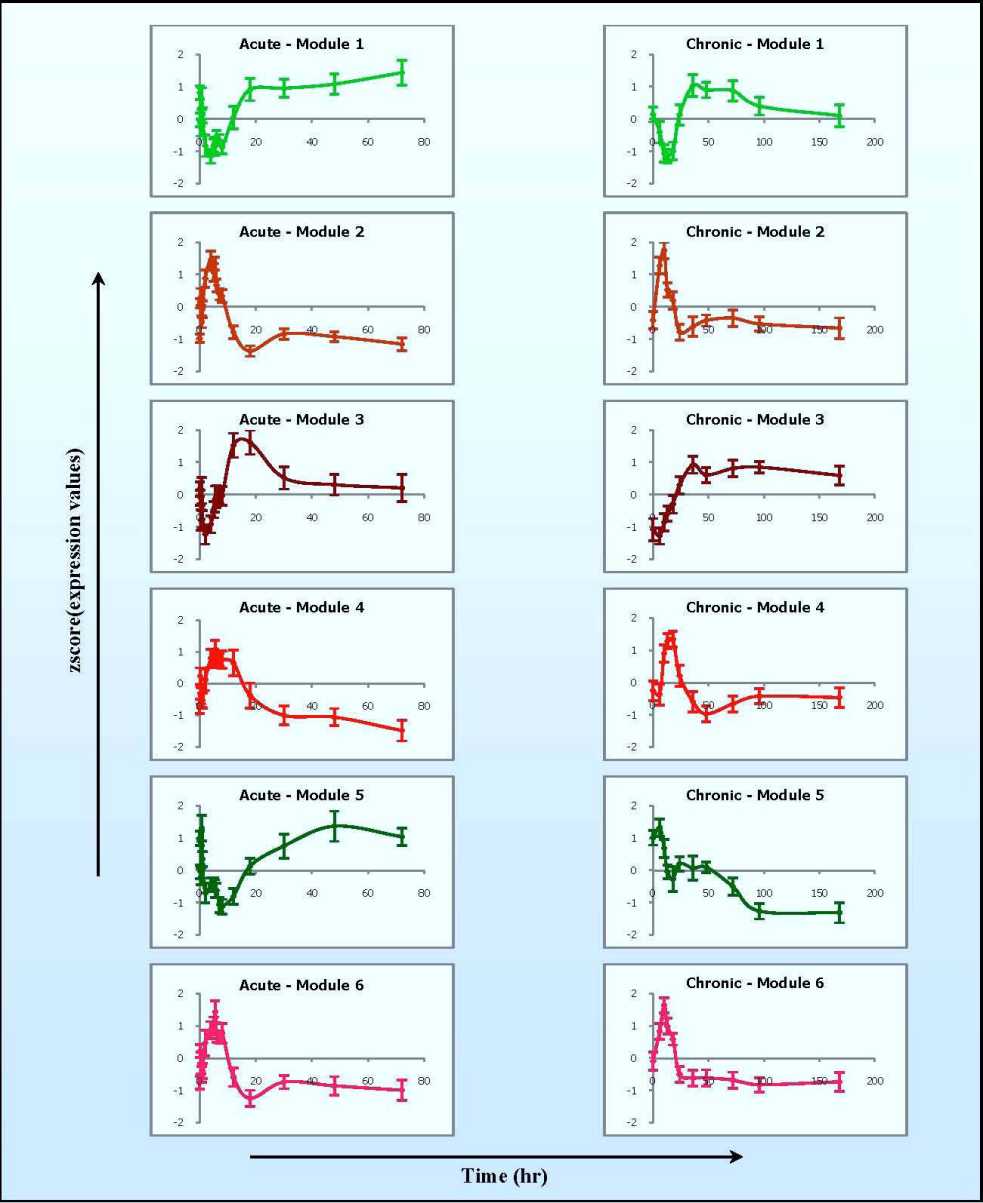
**Critical transcriptional modules of CS pharmacogenomic effects**. Each module is characterized by the average gene expression profile of the corresponding cluster in the acute and the chronic data. The error bar shows the standard deviation of all probeset transcript levels at each time-point in each corresponding pattern.

A detailed description for patterns of these transcriptional modules is shown in Figure 6 with the average expression patterns of all probesets clustered in each module following acute and chronic dosing. In brief, transcriptional module 1 (97 genes) is characterized by one-phase regulation in acute but two phases in chronic dosing. Genes in this module exhibited a fast and robust decline in mRNA, which reached its peak between 4 h and 8 h, and returned to the baseline after about 18 h. However, when MPL is infused (chronic dosing) this set of genes shows a more complex pattern involving both enhanced and suppressed regulation. Although a strong down regulation is observed at the beginning, it is subsequently followed by a sharp induction with the maximum around 36 h and then gradually returned to the baseline indicating some kind of possible tolerance. The second transcriptional module (45 genes) shows a similar pattern of expression in both acute and chronic regimen with two phases of regulation. Genes in this module exhibit an early up-regulation and reached their corresponding peaks at around 4 h in the acute and 10 h in the chronic. Subsequently, both profiles denote a clear down-regulation (around 18 h in acute and 24 h in chronic) and possible slight fluctuation before returning to base line. An interesting dynamics is observed in the 34 genes of transcriptional module 3. In the acute dosing, the genes in this module clearly exhibit an expression pattern with two phases of regulation (down-up-down). Yet, in chronic administration they exhibited an early transient decline in mRNA followed by robust, sustained, up-regulation.

Similar to module 2 is the transcriptional dynamics exhibited by transcriptional module 4 (71 genes) characterized by an early induction with a maximum at 5.5 h in the acute and 18 h in the chronic. A typical pattern with down regulation for both acute and chronic administration is illustrated by transcriptional module 5 (14 genes). However, genes in the acute regimen exhibited a fluctuated repression with a maximum at around 8 h and then followed by an induction to return to the baseline as late as 72 h. Meanwhile, genes in the chronic regimen characterized a pattern with a slightly transient up-regulation followed by a sustained down-regulation and eventual convergence to a new steady state in the presence of the drug. The last transcriptional module (54 genes) has a similar acute pattern of expression with two phases of regulation as that of module 2. However, in the chronic regimen after falling to a value below the baseline (~24 h) this set of genes was further sustained a slight suppression.

While comparing these expression patterns, we observe that modules 2, 4, and 6 have similar expression patterns in acute (2 & 6) or chronic (2 & 4) with a slight difference in the other dosing regimen (e.g. 2 & 6 in chronic, 2 & 4 in acute). Although the difference is not large enough to be intuitively recognized, the merging process could not merge them together, implying that the difference is significant. Furthermore, the separation of these expression patterns is also reinforced with different functional characteristics which will be illustrated below. In summary, selected transcriptional modules exhibit a number of typical expression patterns under corticosteroid administration. The pattern can be simply expressed as an up- or down- regulation or as a more complex one with two phases of regulation plus some fluctuation (see expression patterns in Additional File 2).

### Putative transcriptional regulators of critical transcriptional modules

It has been widely accepted that after corticosteroids bind to cytosolic glucocorticoid receptors (GR), the activated steroid-receptor complex is rapidly translocated into the nucleus where it can alter the expression of target genes. However, the drug seems to be cleared within about 6 h following a bolus injection, suggesting that the mRNA levels of CS-target genes will return to the base line after that [11]. In the contrast, the drug will reach and remain to a stable steady state after 6 h in the chronic administration. Yet, the GR is greatly diminished in response to corticosteroids [14,15,19,20], suggesting that the mRNA levels of CS-target genes in the chronic regimen should also return to the base line. This mechanism is corresponding to the first-phase regulation of target genes. However, almost all chronic patterns involve two phases of regulation and some (module 3 & 5) are only half-phase patterns i.e. persistent up or down without returning to the baseline. These complexities in expression patterns of CS-target genes can be explained by a number of possibilities previous studies have shown [11,12], including multiple GR isoforms, multiple GREs with different affinities to the drug receptor complex, or some other receptors that can mediate the effect of corticosteroids and thus affected genes in this case can reach a new steady state in the presence of the drug (e.g. module 3 & 5).

However, another possibility is a mechanism that results in the regulation of secondary biosignals which transcription factors are the most potential factors. After affected by corticosteroids, they in turn further modulate the expression of glucocorticoid-regulated genes as a continuing cascade of events that were initiated by the drug. As a result, this possibility suggests a possible interpretation of the complexities in expression changes of multiple CS-target genes with the second phase of regulation (e.g. module 1, 2, 4, and 6). In order to reveal some underlying regulatory mechanism of these selected transcriptional modules, we start analyzing the promoter regions of genes to search for significant putative transcriptional regulators as well as possible relationships of regulation. The hypothesis we explore here is that if two or more genes have similar temporal profiles in response to multiple dosing regimens, they are more likely to share some common regulatory mechanisms.

**Figure 7:**
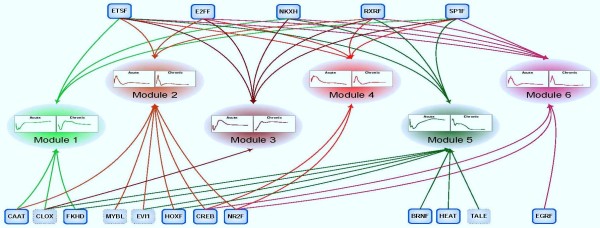
**Putative regulation of CS transcriptional modules by enriched TFBSs**. Those TF families with '**blue**' border lines consist of transcription factors that are affected under corticosteroid administration in this study. The results show a putatively dynamic perspective of regulation between transcriptional regulators and involved sets of genes.

For the 315 genes in six transcriptional modules, we extract 817 *Rattus norvegicus*'s promoter sequences, of which we only keep 194 genes with 502 promoters that include sufficient information of orthologous promoters for further analysis. Figure 7 shows the identified putative regulation between TF families and transcriptional modules. This finding highlights the possibility that secondary biosignals are involved in the regulatory complexities of expression changes for CS-affected genes. Almost all suggested TF families do consist of transcription factor members that are recognized as differentially expressed genes in one or both dosing regimens (see functional characterization in Additional File 3). Since transcription factors are characterized by pleiotropic effects, it is reasonable to observe a significant overlap across various transcriptional modules [75]. While comparing these regulatory combinations, we observe that some TF families seem to be common regulators for all modules (on the top of the figure) whereas some are very specific to particular modules (in the bottom of the figure). This could possibly explain the difference among the expression patterns of these modules. It is likely that the more similar the expression pattern of clusters the more likely they share a larger fraction of common regulators, e.g. TF families in this case. For example, there are a large number of transcriptional regulators that are common between modules 2, 4 and 6 but it seems little overlap exists between the transcriptional regulators of modules 1 and 4, 1 and 6, 2 and 3, except common regulators on the top of the figure.

**Figure 8:**
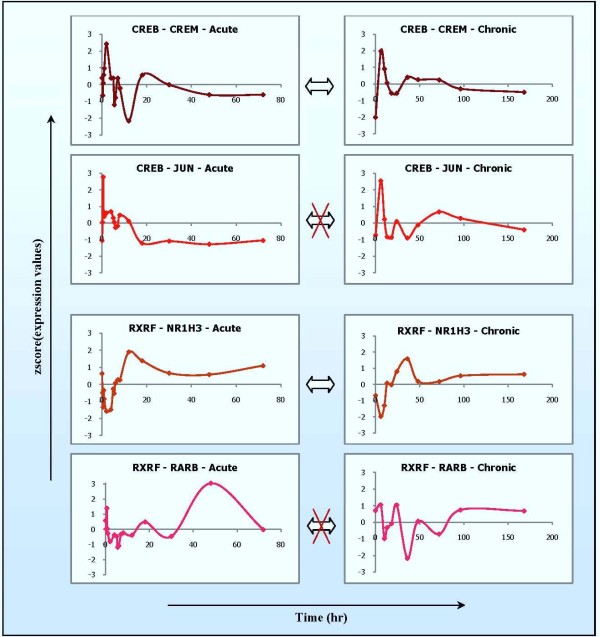
**Expression patterns of several TF representatives in CREB and RXRF family**. One TF family may consist of several members with different expression patterns. One specific TF can have different expression patterns under different conditions (e.g. dosing regimens of corticosteroid administration in this case).

Furthermore, one TF family consists of several TF members whose expression patterns may be similar or different across multiple dosing regimens. Figure 8 shows two examples (CREB family and RXRF family), each of which is represented by two TF members whose expression patterns are similar (CREB-CREM, RXRF-NR1H3) or different (CREB-JUN, RXRF-RARB) between the acute and the chronic corticosteroid administration. Provided that theses TFs are directly involved in the regulation of module 2, 4, and 6, their combinations can produce different ways of regulation, leading to different patterns of expression on target genes among different modules and even in a module but different dosing regimens. As a result, such observations provide a complex, but insightful, perspective into the regulation of these transcriptional modules or the complexities of their expression patterns across multiple dosing regimens.

However, it has been widely recognized that genes affected by CS include both immunosuppressive genes and metabolic genes. Upon the identification of putative transcriptional regulators, their relevance to immune response is demonstrated based on current literature evidence. Specifically, nine among the 29 recognized ETS transcription factors are known to regulate genes involved in immunity [76]; forkhead transcription factors (FKHD) play a major role in the control of apoptosis [77]; and especially CREB has been showed as an essential factor for interactions of glucocorticoid receptors to mediate gene expression [78,79]. A number of others are overlapped with earlier *in silico *studies e.g. E2FF, EGRF, HOXF, NKXH, SP1F [80]. However, given that the experiment of corticosteroid administration has been studied on normal rats, the relevance to adverse effects may be more important than the relevance to immune response. In fact, almost all enriched functions (gene ontologies, pathways) in these transcriptional modules are relevant to metabolic side-effects (see discussion below). Also, due to this reason NFkB and Ap-1 families widely considered as factors involved in inflammation are not present as direct transcriptional regulators for these sets of genes. Furthermore, we identify a number of transcriptional regulators known to be critical factors in metabolic syndrome including obesity, dyslipidemia, hypertension, insulin resistance, etc. e.g. RXRF [81], FKHD [82], SP1F [83]. For instance, the deletion of RXR in mouse liver results in abnormalities of all metabolic pathways regulated by retinoid X receptors heterodimers [84]; FoxOs, members of FKHD family, are able to increase hepatic glucose production, decrease insulin secretion, and affect glucose or lipid metabolism [82].

### Functional characterization of critical transcriptional modules

Since selected transcriptional modules consist of sets of genes that are coexpressed across all dosing regimens, we hypothesize that these genes are more likely involved in critical functions following the drug treatment. Consequently, we search for enriched functions in these modules to explore the functional effects of corticosteroids on target genes as well as evaluate the importance of the selected modules. Using ArrayTrack [85], we first identify the gene ontology terms (GO) that are significant in each transcriptional module (p-value < 0.0001, at least 5 genes). We then classify them into super-categories (so-called main functions) based on the branch of molecular function and biological process in the GO tree. Table 4 lists the distribution of main functions across selected transcriptional modules. In general, all modules are involved in metabolic processes and binding category (except module 5 since it is too small to include significant GO terms). Some modules seem to share almost all main functions e.g. module 2, 4 and 6 whereas others seem to share less e.g. module 2 and 3, 3 and 4, or 3 and 6. However, they are shown to have different roles with specific functions in those main categories. For example although module 2 and 4 are involved in metabolic processes and binding, module 2 is associated with RNAs and nucleotides whereas module 4 is specialized in proteins and macro-molecules. These functional differences (coupled with pathway analysis in Table 5) can be linked to the similarities/differences in their corresponding expression patterns, strengthening the phenomenon that they are classified as distinct transcriptional modules although their expression patterns are not intuitively separated. However, the most important conclusion drawn from this analysis is that all these transcriptional modules consist of components that participate in metabolic processes, implying that they include genes that experience metabolic effects under corticosteroid administration.

**Table 4:** Connecting CS transcriptional modules to enriched gene ontology terms (p-value < 0.0001)

**No**.	Gene Ontology Terms*		Module 1	Module 2	Module 3	Module 4	Module 5	Module 6
1	Metabolic process	Amino acid, compound, organic acid	X					
		
		mRNA		X				
		
		Nucleotide, nucleoside			X			
		
		Protein, macromolecule				X		X

2	Binding	Cofactor, coenzyme, vitamin, heme, ion	X					
		
		Nucleotide, nucleic acid binding		X				
		
		RNA binding						X
		
		Protein binding				X		X

3	Cellular catabolic process		X					

4	Catalytic, oxidoreductase activity		X		X			

5	Oxidative phosphorylation				X			

6	Transmembrane transporter activity				X			

7	Protein-RNA complex assembly			X				

8	RNA splicing, processing			X				

9	Gene expression			X		X		X

10	Translation activity			X		X		

11	Biosynthetic process					X		

12	Structural molecule activity					X		

*: Details are listed in functional characterization in Additional File 3.

**Table 5:** Connecting CS transcriptional modules to enriched biological pathways (p-value < 0.01)

Transcriptional modules	Enriched biological pathways	p-value	**GRE**^**+**^
1	Nitrogen metabolism(rno00910)	0.0000313	X
	Glycine, serine and threonine metabolism(rno00260)	0.0006195	x
	Bisphenol A degradation(rno00363)	0.0009858	√
	Tryptophan metabolism(rno00380)	0.0013596	x
	Histidine metabolism(rno00340)	0.0017470	√
	beta-Alanine metabolism(rno00410)	0.0020365	√
	Bile acid biosynthesis(rno00120)	0.0027013	√
	Arachidonic acid metabolism(rno00590)	0.0053445	
	Pantothenate and CoA biosynthesis(rno00770)	0.0056735	√
	Butanoate metabolism(rno00650)	0.0072639	√
	Tyrosine metabolism(rno00350)	0.0079428	√
	Valine, leucine and isoleucine degradation(rno00280)	0.0094101	√

2	Tyrosine metabolism(rno00350)	0.0000590	√
	Aminophosphonate metabolism(rno00440)	0.0001267	x
	Selenoamino acid metabolism(rno00450)	0.0004668	x
	Histidine metabolism(rno00340)	0.0010152	x
	Alanine and aspartate metabolism(rno00252)	0.0013658	√
	Arginine and proline metabolism(rno00330)	0.0019112	√
	Tryptophan metabolism(rno00380)	0.0040672	x
	Androgen and estrogen metabolism(rno00150)	0.0042813	X

3	Oxidative phosphorylation(rno00190)	9.000E-08	x
	Androgen and estrogen metabolism(rno00150)	0.0000888	
	Starch and sucrose metabolism(rno00500)	0.0020632	
	Urea cycle and metabolism of amino groups(rno00220)	0.0069082	
	Pentose and glucuronate interconversions(rno00040)	0.0076060	

4	Ribosome(rno03010)	0.000E+00	
	Proteasome(rno03050)	0.0000037	X

5	None		

6	Proteasome(rno03050)	2.570E-04	x
	Tight junction(rno04530)	3.410E-04	x
	Long-term depression(rno04730)	4.090E-04	x
	TGF-beta signaling pathway(rno04350)	5.040E-04	x
	Wnt signaling pathway(rno04310)	0.0032164	X

^+^: Glucocorticoid Receptor Element - GRE binding sites; √: GRE binding sites are present on the promoters of almost all genes in the corresponding function group; x: possibly because of not enough promoter information to be considered. Details are listed in functional characterization in Additional File 3.

Using ArrayTrack, we also searched for enriched pathways in these transcriptional modules (p-value < 0.01). A large proportion of significant pathways selected in each module are metabolic pathways of amino acid metabolism or biosynthesis, providing another support that selected transcriptional modules are critical and able to capture metabolic side effects for further analysis. Table 5 shows significant pathways in each transcription module.

It is generally accepted that expression levels of many CS-affected genes are mediated through the binding motifs, called GREs - glucocorticoid response elements, on their control regions. We thus examine the presence of this binding site on the promoter of genes in each of the enriched pathways in order to assess the possible effect of GRE of metabolic functions. However, such GREs are short (5-9 bp) and fairly degenerate, leading to matches occurring by chance alone thus not implying any kind of functionality. In order to address this issue, after extracting gene promoters from the Genomatix database we identified conserved regions across sets of orthologous promoters. As a result, those matches located on these conserved regions would be more reliable estimates of functional binding sites.

Although it is currently believed that GREs are composed of two hexamers with a three-nucleotide random-hinge region in between, the general consensus is that towards one hexamer, namely TGTTCT [10]. We therefore search for this motif on conserved promoter regions across orthologous promoters of the selected genes. The results are shown in Table 5 and detailed information is provided in additional files in functional characterization in Additional File 3. In general, almost all metabolic pathways contain genes with the GRE binding sites, implying that these genes are more likely to be directly regulated by the complex between corticosteroids and glucocorticoid receptors. Additionally, we also examine how frequently the GRE binding sites are present on the control regions of all selected genes (315 genes). Furthermore, we determined that given a background set of 2,000 randomly selected genes, the frequency of GREs in a set of genes is similar to that in the random set (~20%), implying that not all genes in those modules are directly regulated by the drug and that the presence of GRE binding sites on the control regions of genes in enriched pathways is very significant and not random.

## Conclusions

In summary, we have proposed a systematic computational approach that can identify critical transcriptional modules coupled with their common regulatory controls under the CS administration. The approach provides a framework to handle challenging issues related to different platforms, time-grids, genes with multiple probesets, and also different tissues if applicable. Even if the datasets across multiple conditions are present on the same platform, time-grid and tissue, the approach is still useful since genes contain multiple probesets and estimation of a single gene profile by taking the average across these probesets may lose some useful information. However, the analysis may be limited due to the small common set of genes across different platforms.

The computational effectiveness of the approach has been demonstrated on synthetic data. When applying to real time-series datasets, the approach not only yields critical transcriptional modules but also provides an insight into the complexities of regulation of expression patterns. These complexities are further analyzed by techniques in promoter analysis and functional analysis to deduce useful information of transcriptional regulators and enriched metabolic pathways, providing a better understanding towards regulatory mechanisms and adverse pharmacogenomic effects of corticosteroids.

## Authors' contributions

TTN designed the algorithms and experiments, devised and implemented the algorithms. RRA, DCD and WJJ reviewed the material and contributed to the discussion. IPA organized the activities and structured the approach. All authors read and approved the final manuscript.

## Supplementary Material

Additional file 1 **Provide links to sources of synthetic datasets used in this study**. Click here for file

Additional file 2 **Provide detailed results of six significant transcriptional modules, including gene ids, cluster ids, probeset ids and corresponding 'true' expression profiles of identified probesets in response to acute and chronic corticosteroid administration respectively**. Click here for file

Additional file 3 **Provide detailed results of functional analysis, including gene ontology, pathway enrichment, binding information, and corresponding expression profiles of transcription factors found in the study**. Click here for file
