# Supplementary material for: Comparative analysis of acute and chronic corticosteroid pharmacogenomic effects in rat liver: Transcriptional dynamics and regulatory structures
Source: BMC Bioinformatics. 2010 Oct 14;11:515. doi: 10.1186/1471-2105-11-515 (PMC2973961; doi:10.1186/1471-2105-11-515)
Supplement: Additional file 1 — Provide links to sources of synthetic datasets used in this study. [file 1471-2105-11-515-S1.DOC]

**Synthetic data**

<http://expression.microslu.washington.edu/expression/kayee/medvedovic2003/medvedovic_bioinf2003.html>

(Medvedovic M, Yeung KY, Bumgarner RE. (2004) Bayesian mixture model based clustering of replicated microarray data. **Bioinformatics**, 20(8):1222-32)

- Dataset 1

<http://expression.microslu.washington.edu/expression/kayee/medvedovic2003/syn/1rep_high_noise.tar.gz>

- Dataset 2

<http://expression.microslu.washington.edu/expression/kayee/medvedovic2003/syn/3rep_high_noise.tar.gz>

<http://expression.microslu.washington.edu/expression/kayee/cluster2003/yeunggb2003.html>

(Yeung KY, Medvedovic M, Bumgarner RE. (2003) Clustering gene expression data with repeated measurements. **Genome Biology**, 4(5):R34)

- Dataset 3:

<http://expression.microslu.washington.edu/expression/kayee/cluster2003/4rep_high_noise.tar.gz>

- Dataset 4:

<http://expression.microslu.washington.edu/expression/kayee/cluster2003/20rep_high_noise.tar.gz>
